# Seroprevalence of Toxoplasma, Rubella, and Cytomegalovirus among pregnant women in Van

**DOI:** 10.4274/tjod.35902

**Published:** 2015-06-15

**Authors:** Mehmet Parlak, Numan Çim, Begüm Nalça Erdin, Ayşe Güven, Yasemin Bayram, Recep Yıldızhan

**Affiliations:** 1 Yüzüncü Yıl University Faculty of Medicine, Department of Medical Microbiology, Van, Turkey; 2 Yüzüncü Yıl University Faculty of Medicine, Department of Obstetrics and Gynecology, Van, Turkey; 3 Van Training and Research Hospital, Microbiology Laboratory, Van, Turkey

**Keywords:** Toxoplasma, Rubella, Cytomegalovirus, prevalence, Pregnancy

## Abstract

**Objective:**

To determine the seroprevalence of anti-Toxoplasma, anti-Rubella, and anti-*Cytomegalovirus (CMV)* antibodies among pregnant women receiving prenatal care at Van Training and Research Hospital.

**Materials and Methods:**

In developing countries, various infectious agents encountered in the gestational period are important because they influence both maternal and fetal health. Among these, *Toxoplasma gondii*, Rubella and *CMV* are quite prevalent. In the present study, anti-Toxoplasma, anti-Rubella and anti-*CMV* antibodies were analyzed in the serum samples obtained from women receiving prenatal care at Van Training and Research Hospital between June 2012 and July 2013, and positive serum samples were retrospectively evaluated. Anti-Toxoplasma, anti-Rubella and anti-*CMV* antibodies were analyzed using ELISA with Cobas 4000 e411 (Roche, Germany) and Architect i2000SR (Abbott Diagnostics, Germany) analyzers.

**Results:**

Over the course of the study period, the results of a total of 9809 patients were investigated in terms of anti-Toxoplasma, anti-Rubella, and anti-CMV antibodies. Anti-Toxoplasma, anti-Rubella, and anti-*CMV* IgM and IgG antibody positivity rates were 1.1%, 0.5% and 2.6%, and 37.6%, 86.5% and 100%, respectively.

**Conclusion:**

Anti-Toxoplasma IgG antibody positivity rates determined in the present study were lower as compared with the results of the other studies reported from Turkey. However, *CMV* IgM and IgG antibody positivity rates were be higher as compared with those reported in the literature.

## INTRODUCTION

In the developing countries, various infectious agents encountered during the gestational period are important because they influence both maternal and fetal health. Pregnancy-related physiologic changes suppress immunity for a certain period and thereby enhance susceptibility to infectious agents. Among these, the prevalence of Toxoplasma gondii, Rubella, and *Cytomegalovirus (CMV)* are very high and they may cause congenital malformations in the fetus by crossing the placental barrier^(1)^.

Miscarriage and stillbirth are the most serious consequences of congenital toxoplasmosis. If death does not occur, microcephaly, hydrocephaly, cerebral calcifications, convulsions and psychomotor retardation may develop in the fetus. The disease is milder in fetuses that are infected during later prenatal development. Maternal Rubella leads to massive defects including cardiac and ocular anomalies, deafness, and microcephaly in the fetus in the first trimester^(2)^. Half of the primary *CMV* infections encountered during pregnancy affects the fetus. Jaundice, hepatosplenomegaly, petechial rashes, hemolytic anemia, microcephaly, chorioretinitis, and cerebral calcifications may be seen in infants with fulminant cytomegalic inclusion disease. In Turkey, the rate of seropositivity of Toxoplasmosis IgM and IgG is reported to be 4-11% and 47-54%, respectively. In addition to that, *CMV* and Rubella seropositivity rates are reported to be 55-91% and 65-90%, respectively^(1,3)^.

The present study aimed to determine the seroprevalence of Toxoplasma, Rubella, and *CMV* infections among patients receiving prenatal care at Van Training and Research Hospital.

## MATERIALS AND METHODS

Toxoplasma, Rubella and *CMV* antibodies were analyzed in the serum samples of pregnant women receiving prenatal care in the Department of Gynecology and Obstetrics of Van Training and Research Hospital between June 2012 and July 2013, and the positive serum samples were retrospectively investigated. For this purpose, presence of anti-Toxoplasma IgM, anti-Toxoplasma IgG, anti-Rubella IgM, anti-Rubella IgG, anti-*CMV* IgM, anti-*CMV* IgG, anti-Toxoplasma IgG avidity and anti-*CMV* IgG avidity were investigated. Only the initial results of each patient were taken into account and repeated recordings were excluded.

Blood samples obtained for anti-Toxoplasma, anti-Rubella, and anti-*CMV* antibody screening were centrifuged at 10 000 rpm for 15 min and then analyzed using enzyme-linked immunosorbent assay (ELISA) within 2 hours using Cobas 4000 e411 (Roche, Germany) and Architect i2000SR (Abbott Diagnostics, Germany) analyzers. Toxoplasma IgM values higher than 0.6 ratio and Toxoplasma IgG higher than 3 IU/ml were taken as positive. Rubella IgM values with a ratio above 1.6 and Rubella IgG values above 10 IU/ml were considered as positive. *CMV* IgM values with a ratio greater than 1 and *CMV* IgG values greater than 6 AU/mL were regarded as positive.

Z test was used for the comparison of ratios for categorical variables. The level of statistical significance was considered to be 5% and MINITAB version 14 statistical package was used for analyses. The study was approved by the Yüzüncü Yıl University Faculty of Medicine, Human Ethics Committee (30.01.2014/08).

## RESULTS

Over the course of the study period, the results of a total of 9809 patients were analyzed in terms of anti-Toxoplasma, anti-Rubella, and anti-*CMV* antibodies. The rates of anti-Toxoplasma, anti-Rubella, and anti-*CMV* IgM antibody positivity were 1.1%, 0.5%, and 2.6%, respectively, whereas IgG antibody positivity was 37.6%, 86.5%, and 100%, respectively. Avidity test was performed in the serum samples of 54 patients with positive anti-Toxoplasma IgG antibody results, and 35 (64.8%) of these patients had a high avidity test result. The differences between Toxoplasma, Rubella, and *CMV* IgM seropositivity rates were found to be statistically significant. Similarly, the differences between Toxoplasma, Rubella, and *CMV* IgG, the differences between anti-Toxoplasma IgG avidity, and anti-*CMV* IgG avidity seropositivity rates were determined to be statistically significant (p<0.01). Anti-Toxoplasma IgG avidity was low in 15 patients but was within normal ranges in 4 patients. Anti-*CMV* IgG avidity was high in all serum samples (n=150) analyzed. The rate of anti-Toxoplasma, anti-Rubella, and anti-*CMV* antibody positivity is shown in Table 1.

## DISCUSSION

Although Toxoplasmosis is seen in every region of the world, its incidence is higher in tropical regions as compared with cold and arid regions. Transplacental passage is independent from the severity of maternal infection and it correlates with the gestational age at maternal infection. Screening studies performed for Toxoplasma gondii reveal different results for seropositivity. Sen et al. carried out a study in pregnant women in India and reported the rate of anti-Toxoplasma IgM antibody positivity as 19.4%, whereas it was reported to be 13.1% by Yasodhara et al. in the same region. Al-Hindi et al. found the prevalence to be 7.9% in Palestine and reported that the disease was a public health problem among pregnant women^(4,5,6)^. Vilibic-Cavlek et al. reported the rate of IgM and IgG antibody positivity in Croatia to be 0.25% and 29.1%, respectively^(7)^. Ghazi et al. reported the rate of anti-Toxoplasma IgG antibody positivity as 35.6% in Saudi Arabia^(8)^.

With regard to the studies conducted in Turkey, seropositivity for anti-Toxoplasma IgM and IgG antibodies was found to be 0.4% and 48.3%, respectively in Kocaeli province by Tamer et al.; 0.54% and 52.1%, respectively, in Hatay province by Ocak et al.; 2.5% and 43.9% in Zonguldak province by Aynıoğlu et al.; 0.4% and 23.5% in İstanbul province by Karacan et al.; and 1.4% and 37% in Denizli province by Karabulut et al. In the present study, the rate of seropositivity for anti-Toxoplasma IgM and IgG antibodies was found to be 1.1% and 37.6%, respectively^(9,10,11,12,13)^. In another study, which was conducted in our city in 2009, anti-Toxoplasma IgM and IgG antibody positivity were found to be 0.3% and 36%, respectively^(14)^. The IgM and IgG antibody positivity rates obtained in the present study were close to those found in the studies reported from Turkey.

In order to be protected against disease, hands must be carefully washed after contact with uncooked meat and attention must be paid to avoid contact with cat stool. Cysts in the meat are decomposed by cooking at 56 °C for 15 minutes and freezing at -20 °C^(2)^. Therefore, considering the low socioeconomic level and high meat consumption in our region, the present study restates the importance of prenatal care in terms of Toxoplasma.

Rubella infection, which is prevalent in pediatric age group and has a subclinical course in half of the infected individuals and does not cause complications, may lead to serious damage in the fetus when encountered during pregnancy. Rubella in the first two months of pregnancy poses fetal infection risk in 80%^(3)^. Anti-Rubella IgG antibody positivity rate was reported to be 95.3% by Barreto et al.; 93.3% by Ghazi et al.; 85.9% by Ashrafunnessa Khatun et al.; and 76% by Palihawadana et al. in Turkey, Pehlivan et al. reported the positivity rate to be 93.8% for IgG antibody and 0.6% for concurrent IgM and IgG antibody positivity^(8,15,16,17,18)^. The rate of positivity of IgM and IgG antibodies was reported to be 0.2% and 96.1%, respectively, by Tamer et al.; 0.5% and 95.6, respectively, by Karacan et al.; 1.5% and 93.8, respectively by Aynıoğlu et al.; and 0.54% and 95%, respectively by Ocak et al.^(9,10,11,12)^. Anti-Rubella IgM and IgG antibody positivity rates were found to be 0.3% and 99.5%, respectively, by Efe et al. in Van province^(14)^. In the present study, IgM and IgG antibody positivity for Rubella was 0.5% and 86.5%, respectively, which is consistent with the results of many studies. Risk assessment for congenital Rubella must be carried out meticulously, even though the rate of anti-Rubella IgM antibody positivity appears to be low (in five out of one thousand patients). High rate of IgG antibody positivity was interpreted as an indicator of success of immunization practices, which were strictly carried out as the consequence of congenital Rubella syndrome due to Rubella outbreaks encountered in the past.

Specific antibody positivity for *CMV* shows an increase in newborns and in women of childbearing age. It is reported to be higher in young, unmarried, primiparous women, and women with low socioeconomic status. While seropositivity rates are between 50% and 60% in developed countries, the rates are between 90% and 100% in developing countries Seropositivity rate among Turkish pregnant women is estimated to be 74-91%^(1,3)^. When the studies performed on seropositivity in Turkey were evaluated, it was observed that seropositivity for anti-*CMV* IgM and IgG antibodies was reported to be 0.4% and 94.9%, respectively, by Ocak et al.; 0.4% and 84.4%, respectively, by Karacan et al.; 2% and 91.5%, respectively, by Aynıoğlu et al.; and 0.7% and 96.4%, respectively, by Tamer et al.^(9,10,11,12)^. In our province, anti-*CMV* IgM and IgG antibody levels were reported between 1.7% and 99.5%^(14)^. In the present study, *CMV* IgM and IgG antibody positivity rates were 2.6% and 100%, respectively, which were higher than the rates reported in the literature. During acute *CMV* infection, the virus is secreted in urine and saliva for months. Such high rates are understandable when factors such as crowded living conditions, low socio-economic level, and the inadequate infrastructure of our province are considered.

In recent years, specific IgG avidity tests have been suggested as an appropriate method to make differentiation between acute, recurrent or past infections. These tests have been developed for Toxoplasma gondii, *CMV*, and for many other agents. Low avidity antibody for a pathogenic virus indicates a recent infection, but high avidity excludes an infection within the last 3-4 months^(19,20)^. In the present study, the high avidity detected in all patients screened for anti-*CMV* IgG avidity was considered favorable in terms of reduced risk of congenital infection. It should be kept in mind that risk still exists for anti-Toxoplasma IgG antibody because avidity was not high in all patients. In addition, the avidity test was seen to be used less than expected. Although IgG antibody levels for Toxoplasma and *CMV* were positive in the serum samples of 699 patients, the number of serum samples analyzed for avidity was 204, which indicates the necessity to revise our prenatal care approach.

The limitations of this study; the study does not reflect the entire pregnant women population in the area, and pregnancy outcomes could not assessed due to the absence of a woman who detected positive for IgM and IgG.

In conclusion, the Toxoplasma IgG antibody positivity rate was found low in our province, whereas the *CMV* IgG antibody positivity rate was high. Eating habits, life style, weather conditions, and crowded families are considered to be the reason. It is possible that these rates may increase when the harsh weather conditions that make transportation difficult present, and also the low socioeconomic level of our province should be taken into consideration.

## Figures and Tables

**Table 1:**
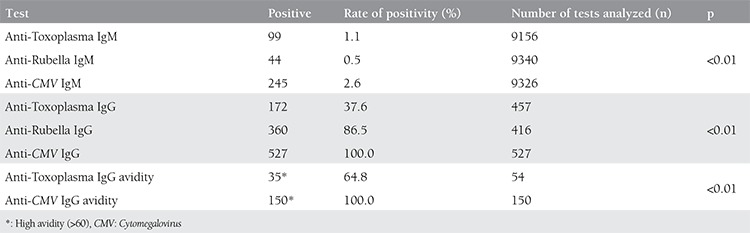
The rates of anti-Toxoplasma, anti-Rubella and anti-Cytomegalovirus antibody positivity
